# FINCHES: A Computational Framework for Predicting Intermolecular Interactions in Intrinsically Disordered Proteins

**DOI:** 10.3390/ijms26136246

**Published:** 2025-06-28

**Authors:** Sarfaraz K. Niazi

**Affiliations:** College of Pharmacy, University of Illinois, Chicago, IL 60612, USA; sniazi3@uic.edu; Tel.: +1-312-297-0000

**Keywords:** FINCHES, intrinsic disorder, proteins, intermolecular interaction, force field, molecular dynamics, AWSEM

## Abstract

This comprehensive review examines FINCHES (Force field-based Interaction Network for Characterizing Heterotypic and Entropic Sequences). This groundbreaking computational framework enables the rapid, sequence-based prediction of intermolecular interactions in intrinsically disordered regions (IDRs) without the need for molecular simulations. The document provides detailed comparisons with other computational methods, including their mathematical foundations, specific applications, and experimental validations. We explore both the potential for advancing our understanding of disordered protein function and the inherent challenges in computationally modeling these dynamic biological systems. Additionally, we discuss computational assessment tools for interface prediction in molecular complexes, providing a comprehensive framework for evaluating IDR interaction predictions.

## 1. Introduction

Intrinsically disordered regions (IDRs) are prevalent in over 70% of human proteins and play crucial roles in cellular processes despite lacking stable three-dimensional structures [1,2]. Understanding how these regions mediate specific intermolecular interactions has been challenging due to their dynamic nature and the complexity of their interaction landscapes [3]. This review examines FINCHES (Force field-based Interaction Network for Characterizing Heterotypic and Entropic Sequences). This recently developed computational framework enables rapid, sequence-based prediction of IDR-mediated interactions without requiring molecular simulations [4]. We discuss the methodology, applications, and limitations of this approach alongside detailed comparisons with other computational methods, including their mathematical foundations, specific applications, and experimental validations [5,6,7]. We highlight both the potential for advancing our understanding of disordered protein function and the inherent challenges in computationally modeling dynamic biological systems [8,9].

Intrinsically disordered proteins and protein regions represent a paradigm shift in our understanding of protein structure–function relationships [1,2]. Unlike globular proteins that fold into stable three-dimensional structures, IDRs exist as dynamic ensembles of conformations, yet they are capable of mediating highly specific biological interactions [1,10]. These regions are particularly enriched in transcription factors, signaling proteins, and other regulatory molecules, where they facilitate complex formation, phase separation, and allosteric regulation [2,11]. The challenge of predicting how these dynamic regions interact with cellular partners has hindered our ability to understand their functional mechanisms and design therapeutic interventions [12,13].

Traditional computational approaches for studying protein interactions rely heavily on molecular dynamics simulations, which, while accurate, are computationally expensive and become prohibitively slow for larger systems or proteome-scale analyses [14,15,16]. The development of coarse-grained force fields specifically parameterized for IDRs has improved the speed and accuracy of such simulations. Still, the need for alternative approaches that can rapidly screen large numbers of sequences and conditions remains pressing [17,18]. FINCHES addresses this need by repurposing established force field equations to make analytical predictions about intermolecular interactions without performing explicit simulations [4].

## 2. Computational Approaches for IDR Interaction Prediction

The prediction of IDR interactions has become a critical challenge in computational biology, leading to the development of diverse methodological approaches [19,20]. These can be broadly categorized into physics-based methods, machine-learning approaches, hybrid techniques, and specialized tools for phase separation prediction [21,22,23]. Each approach offers unique advantages and limitations, making them suitable for different applications and research questions [24,25].

### Physics-Based Approaches

**Figure 1:**
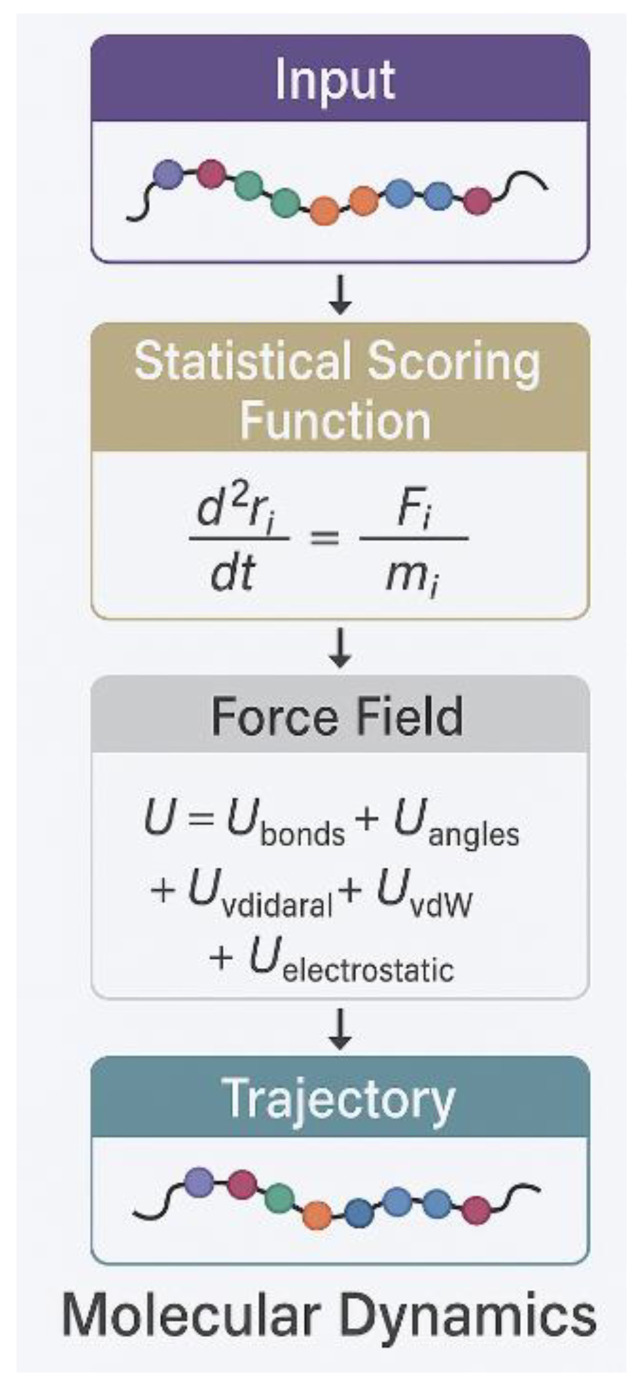
A diagram of a mathematical function of molecular dynamics.

Molecular dynamics simulations remain the gold standard for detailed studies of protein interactions, providing atomistic resolution of interaction mechanisms and dynamics [14,15]. Figure 1 illustrates the fundamental components of molecular dynamics simulations, showing how force field equations govern protein behavior. The fundamental equations governing molecular dynamics (MD) simulations are based on Newton’s equations of motion, where the force F acting on each atom equals mass times acceleration (F = ma) [26]. The force field potential typically includes bonded and non-bonded terms, with the electrostatic component being crucial for IDR studies and calculated using Coulomb’s law: U_elec = k_e × q_i × q_j/r_ij, where k_e represents Coulomb’s constant, q_i and q_j are partial charges on atoms i and j, and r_ij is the distance between them.

All-atom molecular dynamics (MD) simulations can capture the full complexity of IDR behavior, including conformational changes upon binding and the role of water and ions in mediating these interactions [27,28]. However, these simulations are computationally intensive, limiting their application to small systems and short timescales [29]. The timescales accessible to conventional molecular dynamics (MD) simulations (typically microseconds) often fall short of the millisecond-to-second timescales relevant for many biological processes involving intrinsically disordered regions (IDRs) [30].

Shaw et al. [31] utilized the Anton supercomputer to conduct millisecond-scale simulations of small IDR peptides, thereby revealing the role of transient secondary structures in binding. Rauscher et al. [32] investigated the folding mechanism of the p53 transactivation domain upon binding using enhanced sampling molecular dynamics (MD), demonstrating how disorder-to-order transitions facilitate specific recognition. More recently, Palazzesi et al. [33] employed all-atom molecular dynamics (MD) to investigate the phase separation of the FUS protein, revealing that transient π–π interactions between tyrosine residues drive droplet formation.

Enhanced sampling methods, such as replica exchange molecular dynamics (REMD) and metadynamics, have been developed to overcome some of these limitations by improving conformational sampling efficiency [32,33]. REMD uses the exchange principle, where configurations at different temperatures are periodically exchanged based on the Metropolis criterion, allowing the system to overcome energy barriers more effectively [34].

**Figure 2:**
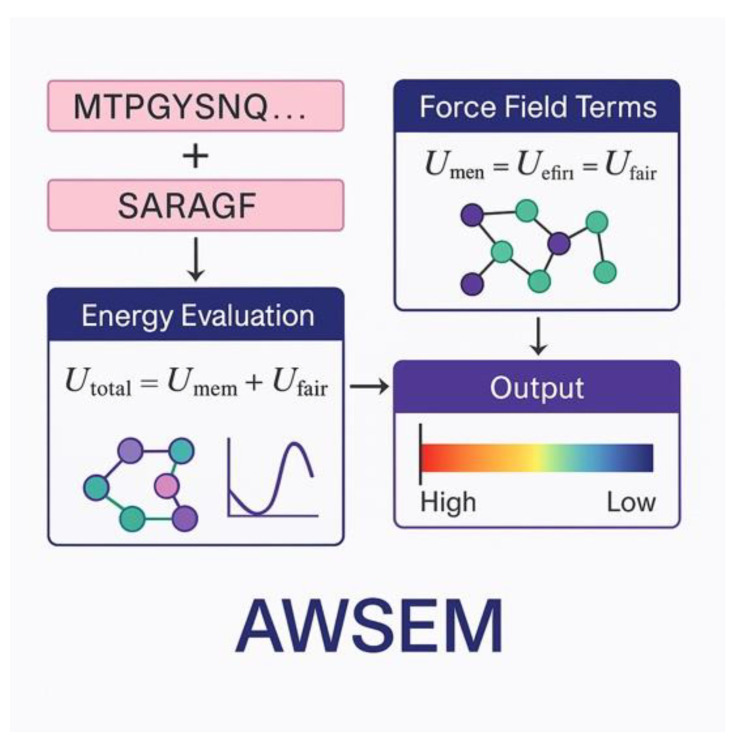
A diagram of energy efficiency AWSEM.

The Associative Memory Water-Mediated Structure and Energy Model (AWSEM) represents a sophisticated, coarse-grained approach that combines knowledge-based potentials with physical principles [35,36]. Figure 2 demonstrates the energy components within the AWSEM framework. The total energy function in AWSEM includes multiple terms representing different aspects of protein energetics: E_total = E_backbone + E_contact + E_burial + E_hydrogen + E_water + E_fragment, where E_backbone accounts for local backbone geometry, E_contact represents residue–residue contact, E_burial accounts for hydrophobic burial, E_hydrogen represents hydrogen bonding, E_water accounts for water-mediated interactions, and E_fragment incorporates local structure biasing from the Protein Data Bank [37].

Chen et al. [38] utilized the AWSEM to investigate the aggregation of α-synuclein, elucidating how specific sequence regions facilitate fibril formation. The simulations accurately predicted the experimental observation that specific mutations can either enhance or suppress the aggregation propensity [39]. Zheng et al. [40] applied AWSEM to investigate the liquid–liquid phase separation of RNA-binding proteins, demonstrating that the model can reproduce experimental phase diagrams when calibrated appropriately.

**Figure 3:**
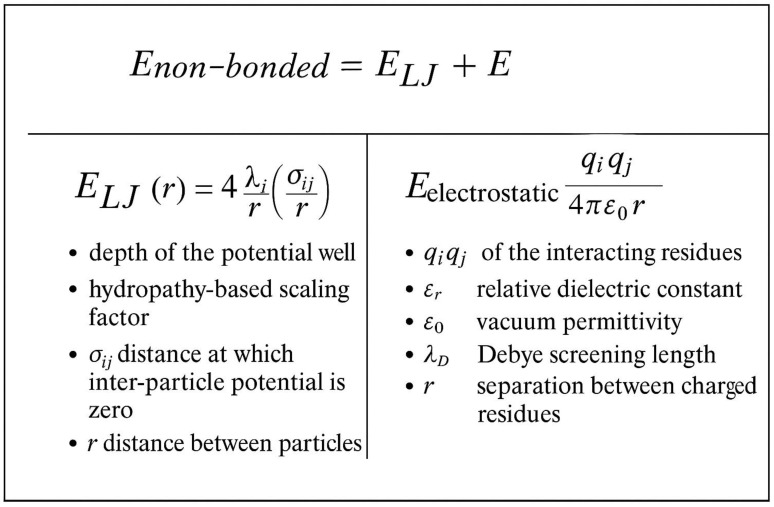
A diagram of Calvados representation.

The CALVADOS force field represents a significant advancement in IDR simulation, specifically designed to reproduce experimental phase behavior [17,41]. Figure 3 illustrates the coarse-grained representation employed by CALVADOS, where each amino acid is represented by a single bead positioned at the Cα atom. The non-bonded energy in CALVADOS combines Lennard–Jones and electrostatic interactions: U_nb = 4ε[(σ/r)^12^ − (σ/r)^6^] + k_e × q_i × q_j × exp (−κr)/r, where ε and σ are Lennard–Jones parameters specific to each amino acid pair, and κ is the Debye screening parameter. Other variables are as defined previously [42].

Tesei et al. [17] validated CALVADOS against experimental data for over 20 different IDR systems, achieving quantitative agreement with the radius of gyration measurements and phase separation temperatures. Krainer et al. [43] utilized CALVADOS to investigate heterotypic interactions between distinct RNA-binding proteins, demonstrating how sequence complementarity drives co-phase separation. The simulations correctly predicted that hnRNPA1 and FUS form mixed condensates, while hnRNPA1 and TDP-43 show limited mixing, consistent with experimental observations [44].

**Figure 4:**
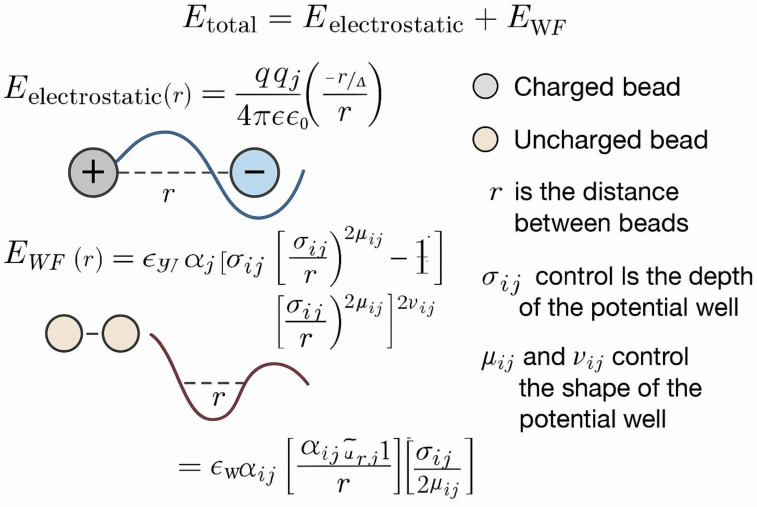
A diagram of Mpipi Force field.

The Mpipi force field employs a minimal, coarse-grained representation, where each amino acid is represented by a single bead [18]. Figure 4 shows the schematic representation of the Mpipi approach. The total energy includes electrostatic and Wang–Frenkel terms: U_total = U_elec + U_WF, where U_elec follows Debye–Hückel screening and U_WF accounts for steric and hydrophobic interactions through a modified Lennard–Jones potential with amino acid-specific parameters [45].

Emenecker et al. [46] employed Mpipi simulations to investigate the relationship between sequence composition and single-chain properties for hundreds of intrinsically disordered protein (IDP) sequences. The force field accurately reproduced the experimental radius of gyration measurements across diverse sequence types [47]. Ginell et al. [48] applied Mpipi to study the effects of post-translational modifications on intrinsically disordered region (IDR) conformations, demonstrating how phosphorylation-induced charge changes alter protein compaction.

**Figure 5:**
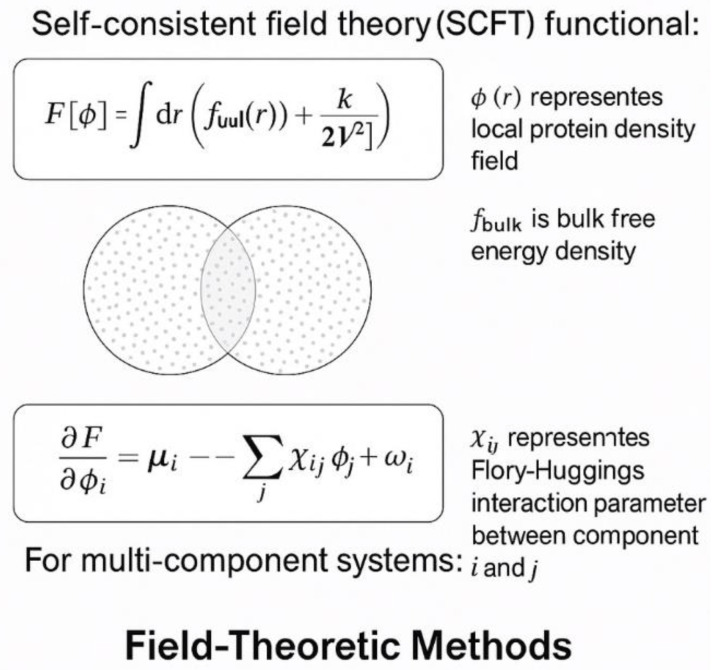
A diagram of field theory methods.

Field-theoretic methods treat proteins as polymers in solution and solve the polymer field theory equations directly [49,50]. Figure 5 illustrates how these approaches model protein systems as continuous fields. The key equation is the self-consistent field theory (SCFT) functional: F[φ] = ∫dr[f_loc(φ(r)) + K/2||∇φ(r)||^2^], where φ(r) is the local density field, f_loc is the local free energy density, K is the gradient penalty parameter, and the integral is over all space. The Flory–Huggins interaction parameter χ_ij describes interactions between components i and j [51,52].

McCarty et al. [53] employed field-theoretic simulations to generate comprehensive phase diagrams for model IDR sequences, revealing how charge patterning influences critical temperatures and concentrations. The approach successfully predicted experimental observations that a uniform charge distribution leads to stronger phase separation than a randomly distributed charge [54]. Danielsen et al. [55] applied SCFT to study the effect of salt concentration on phase behavior, showing quantitative agreement with experimental measurements for several RNA-binding proteins.

## 3. Machine Learning Approaches

**Figure 6:**
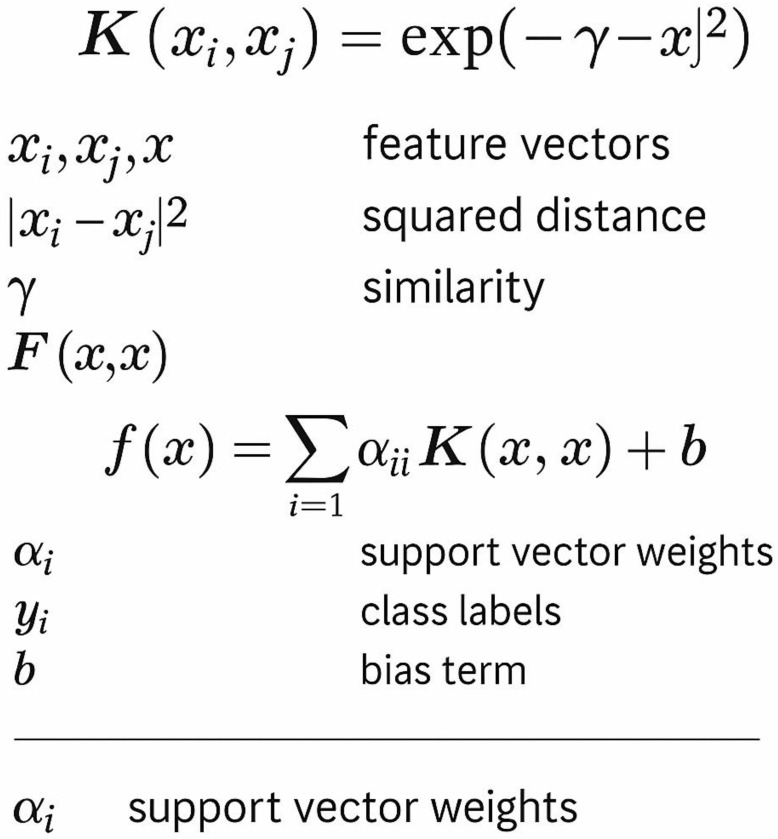
A diagram of PS-Predictor.

The PSPredictor represents one of the first successful applications of machine learning to phase separation prediction [56]. Figure 6 shows the architecture and feature representation used in PSPredictor. The method uses a support vector machine with a radial basis function kernel: K(x_i, x_j) = exp(−γ||x_i − x_j||^2^), where γ is the kernel parameter that controls the kernel width. The feature vector includes amino acid composition (20 features), dipeptide composition (400 features), charge distribution parameters (including fraction of charged residues (FCR), net charge per residue (NCPR), and charge asymmetry parameter (κ)), hydropathy moment calculations, and secondary structure propensities.

Orlando et al. [56] trained PSPredictor on a dataset of 239 experimentally validated phase-separating proteins and 672 negative controls. The method achieved 85% accuracy in cross-validation and successfully predicted phase separation for several previously uncharacterized proteins [57]. Validation studies showed that TIA1, G3BP1, and several other stress granule proteins were correctly identified as phase-separating, while most globular proteins were correctly classified as non-phase-separating [58].

## 4. Deep Learning Approaches

**Figure 7:**
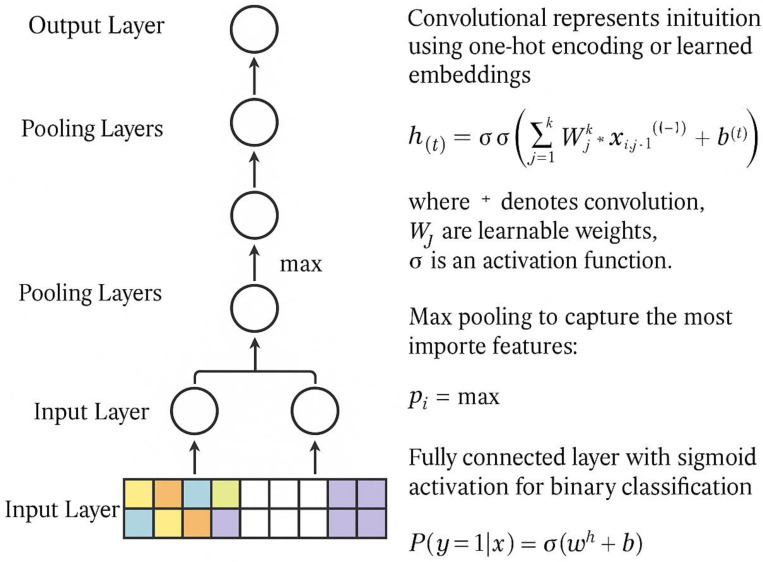
A diagram of the deep learning approach.

Several deep learning architectures have been applied to IDR analysis [59,60]. PSP (Phase Separation Predictor) utilizes a convolutional neural network with a specifically designed architecture for protein sequence analysis [61]. Figure 7 illustrates the deep learning architecture employed for phase separation prediction. The input layer uses one-hot encoding, where each amino acid is represented as a 20-dimensional binary vector or learned embeddings, typically 50–100-dimensional dense vectors that capture the physicochemical similarities of amino acids [62]. The convolutional layers employ multiple filter sizes (three, five, and seven residues) to capture local sequence patterns at different scales, with ReLU activation functions applied as f(x) = max(0,x). Max pooling layers with a stride of 2 reduce dimensionality while preserving important features. The architecture comprises two fully connected layers with 128 and 64 neurons, respectively, utilizing dropout regularization with a probability of 0.5 to prevent overfitting. The output layer contains a single neuron with sigmoid activation for binary classification: P(phase_sep) = 1/(1 + e^−z^), where z is the pre-activation output.

Performance comparisons revealed that one-hot encoding achieved 85% accuracy, while learned embeddings improved performance to 88% accuracy by better capturing the physicochemical similarities of amino acids [63]. The learned embeddings allow the model to understand that chemically similar amino acids (such as leucine and isoleucine) should have identical representations, leading to better generalization across diverse protein sequences.

Mészáros et al. [63] demonstrated that PSP achieves 88% accuracy on a balanced dataset of phase-separating and non-phase-separating proteins. The method correctly identified 23 out of 25 experimentally validated stress granule proteins and exhibited low false-positive rates on globular protein datasets [64]. Feature analysis revealed that the model learned to focus on regions with high aromatic content and specific charge patterns [65].

## 5. Protein Language Model Applications

**Figure 8:**
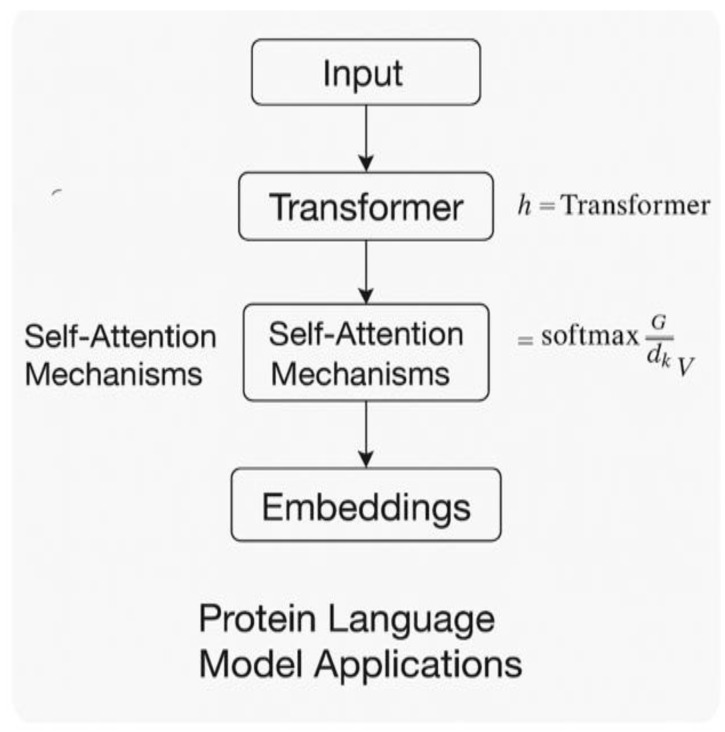
A diagram of the protein language model.

Recent advances in protein language models have been applied to IDR analysis [66,67]. Figure 8 demonstrates how protein language models generate sequence embeddings through transformer architectures. ProtT5 and ESM-1b embeddings capture complex sequence relationships through transformer architectures that employ self-attention mechanisms: Attention(Q, K, V) = softmax(QK^T/√d_k)V, where Q, K, and V are query, key, and value matrices derived from the input sequence representations.

The fine-tuning process for these models involves several carefully orchestrated steps. First, embeddings are extracted from pre-trained models, with ESM-1b providing 1024-dimensional representations for each residue. A task-specific classification head is then added, typically consisting of two layers that reduce dimensionality from 1024 to 256 to 64 neurons, followed by a final classification layer with 2 neurons for binary prediction. Training employs the Adam optimizer with a learning rate of 2 × 10^−5^ and a batch size of 32, often using gradient accumulation to effectively achieve larger batch sizes when memory constraints limit the direct use of larger batches. Early stopping is applied based on validation loss to prevent overfitting and ensure optimal generalization performance. Input sequences are tokenized using model-specific vocabularies and padded to a maximum length of 1024 residues to accommodate the longest proteins in typical datasets while maintaining computational efficiency.

Raimondi et al. [68] used ESM-1b embeddings to predict phase separation, achieving 91% accuracy by combining pre-trained representations with task-specific fine-tuning. The approach successfully predicted that several viral proteins (SARS-CoV-2 nucleocapsid, Ebola VP30) undergo phase separation, which was subsequently confirmed experimentally. The experimental validation employed multiple complementary techniques: turbidity measurements at 350 nm to detect droplet formation and quantify the propensity for phase separation, fluorescence recovery after photobleaching (FRAP) to assess droplet dynamics and internal rearrangement rates, and differential interference contrast (DIC) microscopy to visualize droplet morphology and confirm liquid-like behavior [69].

**Figure 9:**
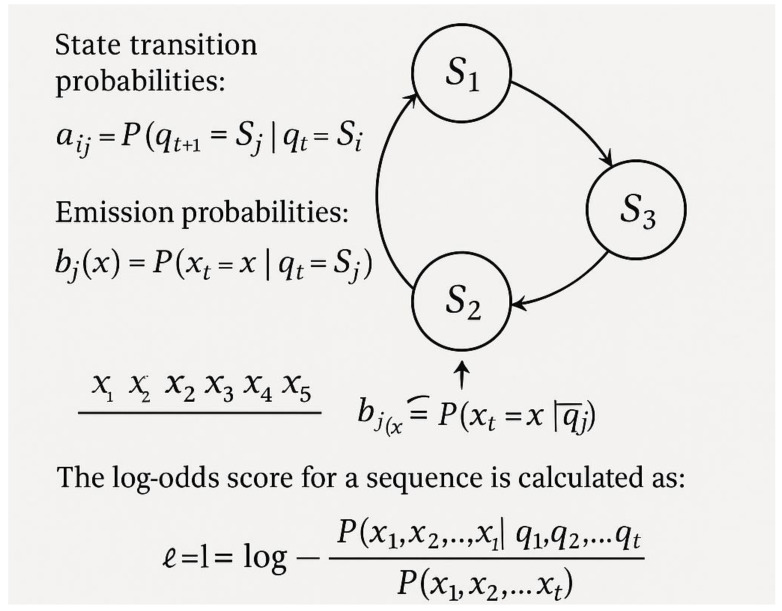
PLAAC modeling.

PLAAC utilizes hidden Markov models specifically designed to identify prion-like domains [70]. Figure 9 illustrates the PLAAC modeling approach, featuring its hidden Markov model architecture. The model defines states representing different amino acid preferences, with a core state that favors prion-like residues such as asparagine, glutamine, serine, and tyrosine, a boundary state with intermediate preferences that allow for transitions between core and background regions, and a background state representing general amino acid composition typical of non-prion domains. State transition probabilities are learned from known prion proteins, with emission probabilities specific to each state optimized for prion domain detection based on the amino acid preferences observed in experimentally validated prion-forming proteins.

Lancaster et al. [70] applied PLAAC to analyze over 200 yeast proteins, identifying 24 proteins with significant prion-like domains. Experimental validation confirmed that 19 of these proteins could form amyloid-like aggregates in vitro, demonstrating the predictive power of the computational approach [71]. Subsequent studies utilized PLAAC to identify prion-like domains in RNA-binding proteins associated with ALS and frontotemporal dementia, revealing potential therapeutic targets and highlighting the pathological relevance of prion-like aggregation in neurodegenerative diseases [72,73].

## 6. FuzDrop: Integrated Disorder and Droplet Prediction

**Figure 10:**
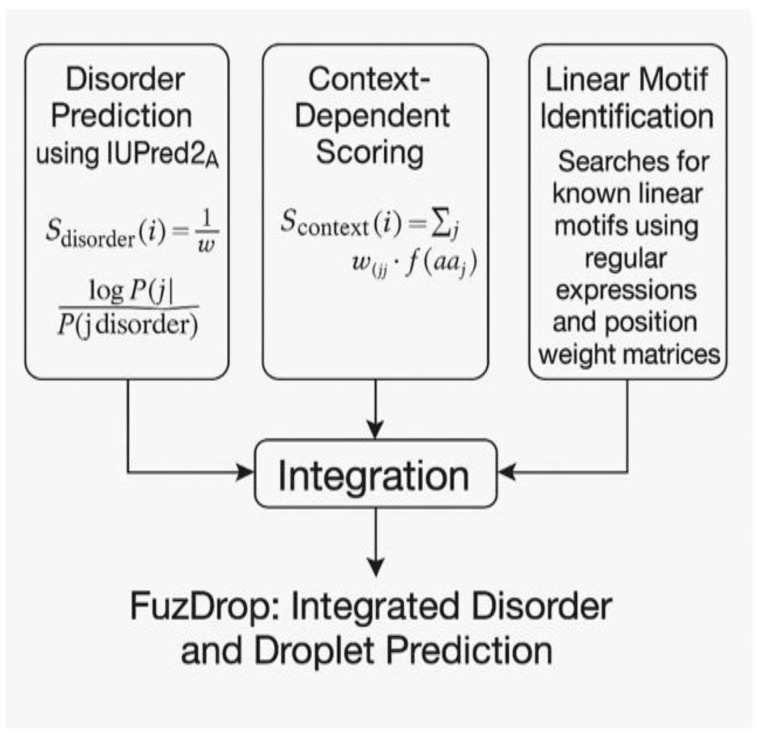
A diagram of FuzDrop.

FuzDrop combines multiple computational approaches to predict droplet-forming regions [74,75]. Figure 10 illustrates the integrated prediction system employed by FuzDrop. The method integrates disorder prediction using IUPred2A scores to identify disordered regions that are prerequisites for droplet formation, compositional analysis that calculates amino acid composition biases toward low-complexity sequences characteristic of phase-separating proteins, and linear motif identification that searches for known linear motifs using regular expressions and position weight matrices derived from experimentally characterized droplet-forming proteins [76].

The determination of sensitivity and specificity thresholds employed rigorous ROC curve analysis on a carefully curated training set of 150 validated droplet-forming proteins. The optimal threshold (score > 0.6) was selected to maximize the F1 score, which is calculated as F1 = 2 × (precision × recall)/(precision + recall), resulting in 92% sensitivity and 73% specificity. This threshold selection process involved the systematic evaluation of multiple cutoff values to identify the optimal balance between sensitivity and specificity for practical applications. Proteins with scores between 0.5 and 0.7 represent borderline cases, where experimental validation is particularly valuable, as these may indicate context-dependent droplet formation or proteins that require specific cofactors for phase separation. Examples of borderline cases include proteins that form droplets only under particular stress conditions or in the presence of RNA cofactors.

Statistical analysis of the correlation between predicted scores and experimental critical concentrations employed Spearman correlation analysis, yielding a correlation coefficient of ρ = 0.67 with *p* < 0.001, indicating a significant but moderate correlation. This correlation suggests that while FuzDrop scores correlate with experimental phase separation propensity, additional factors such as protein concentration, temperature, ionic strength, and cellular environment significantly influence critical concentrations and must be considered when interpreting predictions.

Erdős and Dosztányi [74] tested FuzDrop on 246 experimentally validated droplet-forming proteins, achieving 92% sensitivity and 73% specificity. The method correctly identified droplet-forming regions in p53, c-Myc, and several RNA-binding proteins [77]. Comparisons with experimental data showed a strong correlation between predicted scores and experimentally determined critical concentrations [78].

## 7. catGranule: Machine Learning for Stress Granule Proteins

**Figure 11:**
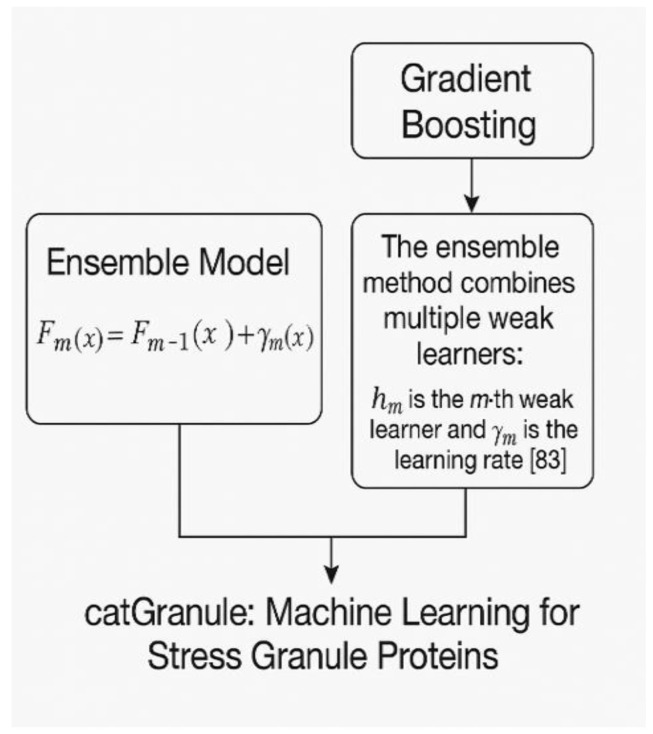
A diagram of catGranule.

catGranule uses gradient boosting to predict stress granule localization [79]. Figure 11 demonstrates the gradient-boosting ensemble approach. The ensemble method combines multiple weak learners (decision trees) according to the formula: F(x) = Σ(i = 1 to M) α_i × h_i(x), where h_i are weak learners, α_i are weights determined during training through the boosting algorithm, and M is the number of boosting iterations. Each decision tree learns from the residuals of previous predictions, progressively improving the overall model performance by focusing on examples that were previously misclassified.

Klim et al. [79] achieved 89% accuracy on a dataset of 570 stress granule proteins and 1140 controls. The method correctly predicted stress granule localization for TIA1, G3BP1, and CAPRIN1 and identified novel candidates that were subsequently validated experimentally through fluorescence microscopy and immunofluorescence staining [80].

## 8. LLPSDB and Database-Driven Approaches

**Figure 12:**
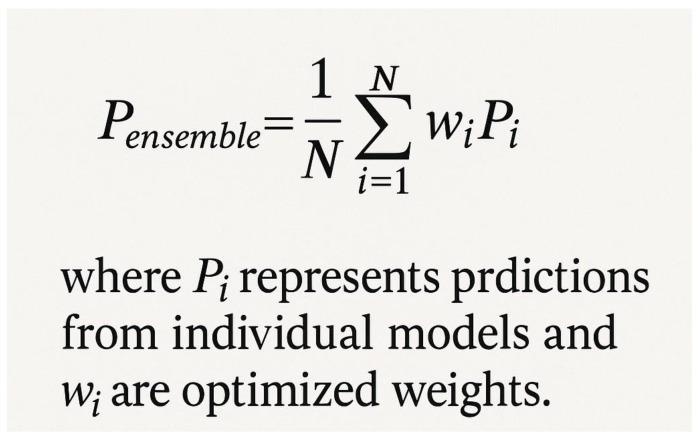
LLPSDB data-driven model.

The Liquid–Liquid Phase Separation Database (LLPSDB) has enabled systematic computational approaches [81]. Figure 12 shows the database-driven modeling approach that leverages curated experimental data. The database contains 1522 phase-separating proteins from 31 species, including experimental conditions such as temperature, pH, and salt concentration ranges that enable phase separation. It also provides structural and functional annotations that offer context for understanding phase separation mechanisms, as well as comprehensive literature references that facilitate detailed validation of predictions. Database-driven predictors use this curated information to train more robust models that can generalize across diverse protein families and experimental conditions [82]. The LLPS-Pred ensemble method combines multiple algorithms (Support Vector Machines, Random Forest, and Neural Networks) with weighted voting: P_final = Σ(i = 1 to N) w_i × P_i, where w_i are algorithm weights learned during training to optimize overall prediction accuracy and P_i are the individual algorithm predictions.

## 9. Physics-Informed Machine Learning

**Figure 13:**
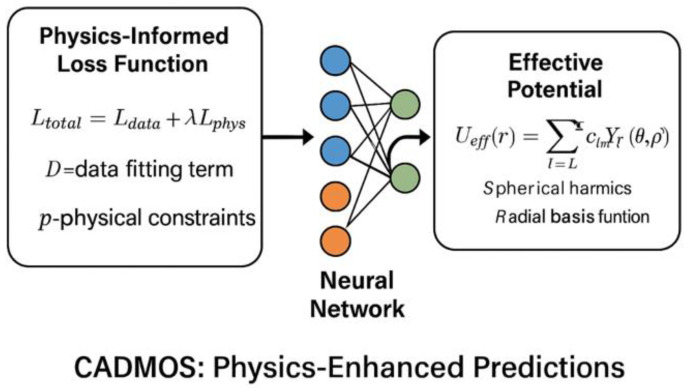
A diagram of CADMOS.

CADMOS combines coarse-grained simulations with neural networks to learn effective potentials [83,84,85]. Figure 13 illustrates the CADMOS physics-enhanced prediction system. The approach uses a physics-informed loss function that incorporates thermodynamic constraints: L_total = L_data + λ × L_physics, where L_data represents the standard data fitting loss that ensures agreement with experimental observations, L_physics ensures predictions satisfy known thermodynamic relationships such as detailed balance and thermodynamic consistency, and λ balances the contributions of both terms to achieve optimal performance (Figure 13).

Maristany et al. [83] applied CADMOS to study FUS phase separation, achieving 95% accuracy in predicting experimental phase diagrams while being 100× faster than traditional simulations. The method revealed that transient contact formation, which is not captured by mean-field theories, is crucial for accurately predicting phase behavior [86].

## 10. Computational Assessment of Interface Prediction

Recent developments in computational assessment tools for interface prediction in molecular complexes provide valuable frameworks for evaluating IDR interaction predictions. These tools are particularly relevant for benchmarking methods like FINCHES against experimental structural data when available, giving standardized metrics for comparing different computational approaches.

DockQv2 represents an advanced scoring system for evaluating the quality of predicted protein–protein interfaces [87]. The method combines multiple geometric and energetic criteria to assess interface accuracy, making it suitable for evaluating FINCHES predictions when experimental complex structures are available. The scoring function incorporates interface contact accuracy by comparing predicted and experimental residue–residue contact, geometric complementarity through shape correlation functions, and energetic favorability by assessing the thermodynamic stability of predicted interfaces. This comprehensive assessment approach provides multiple perspectives on prediction quality, enabling the identification of specific aspects that require improvement.

I-INF provides specialized metrics for assessing interface prediction accuracy, particularly valuable for evaluating the spatial accuracy of predicted interaction regions in IDR complexes [88]. The method focuses on identifying correctly predicted interface residues and quantifying the accuracy of predicted interaction surfaces using precision and recall metrics specifically designed for interface evaluation. This tool is particularly useful for evaluating the spatial resolution of predictions and pinpointing regions where computational methods excel or falter.

I-RMSD offers complementary interface assessment capabilities, focusing on the geometric accuracy of predicted binding sites [89]. This tool calculates root-mean-square deviations between predicted and experimental interface geometries, providing quantitative measures of structural accuracy that can be directly compared across different prediction methods. The approach is particularly valuable for assessing the geometric fidelity of predicted complexes and identifying systematic biases in computational approaches.

These assessment tools could be incorporated into FINCHES benchmarking protocols to provide standardized evaluation metrics for interface prediction accuracy, particularly when validating predictions against available experimental structures of IDR-containing complexes. Such integration would enable the systematic comparison of different computational approaches and provide confidence measures for predictions in the absence of experimental validation. The implementation of these tools would involve establishing standardized protocols for structure preparation, interface definition, and scoring interpretation that ensure consistent and meaningful comparisons across different studies and research groups.

## 11. FINCHES Methodology and Theoretical Foundation

**Figure 14:**
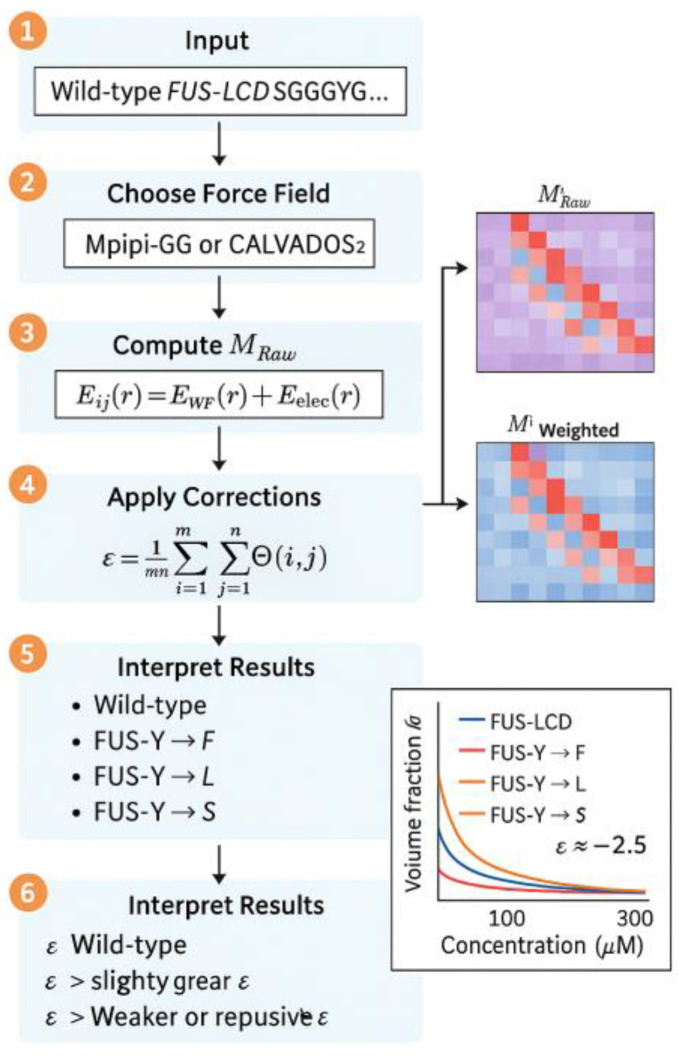
The FINCHES system.

FINCHES operates on the principle that the chemical physics underlying molecular force fields can be leveraged to predict intermolecular interactions analytically [4]. Figure 14 illustrates the comprehensive FINCHES computational framework, showing the sequence-to-interaction pipeline that processes input sequences through force field calculations. The framework uses established force fields, primarily Mpipi-GG and CALVADOS2, which have been extensively validated for describing IDR behavior [17,18]. The fundamental approach involves integrating force field functions over relevant distance ranges to extract effective interaction strengths that quantify the overall attractive or repulsive nature of interactions between residue types. This integration yields a mean-field parameter that captures the essential thermodynamics of protein interactions without requiring explicit simulations, enabling the rapid screening of large numbers of sequences and conditions (Figure 14).

### 11.1. Force Field Implementation and Selection Rationale

The framework implements two primary force fields with distinct advantages and specific application domains [90]. The Mpipi-GG force field combines electrostatic interactions calculated using Coulomb’s law with Debye–Hückel screening and Wang–Frenkel contributions that capture steric and hydrophobic effects. The electrostatic component includes salt-dependent screening effects, making it particularly suitable for studying how ionic strength influences IDR interactions [91,92]. This force field is preferentially applied to salt-dependent analyses within the ionic strength range of 0.01–1.0 M to systems with significant charge–charge interactions where electrostatic effects dominate the interaction landscape, and to studies focusing specifically on electrostatic contributions to binding, where a detailed understanding of charge-mediated interactions is required.

CALVADOS2 uses a different functional form but captures similar physical principles, employing temperature-dependent dielectric constants and Yukawa potentials for electrostatic screening [17]. This force field is preferentially applied to temperature-dependent studies within the physiological range of 285–310 K, phase separation investigations where accurate reproduction of experimental phase diagrams is critical, systems where hydrophobic interactions dominate the interaction landscape, and multi-component mixture analyses where multiple protein species interact simultaneously. The temperature dependence in CALVADOS2 makes it particularly suitable for studying thermal stability and temperature-induced phase transitions.

The selection between force fields follows specific guidelines based on experimental conditions and system characteristics. CALVADOS2 is recommended when experimental data indicates strong temperature dependence or when studying phase separation phenomena where accurate, critical temperature prediction is essential. Mpipi-GG is preferred for salt titration studies or when electrostatic interactions are expected to dominate based on sequence composition (high charge content) or experimental observations (strong ionic strength dependence). For systems where both temperature and salt effects are significant, a comparative analysis using both force fields can provide valuable insights into the dominant interaction mechanisms.

### 11.2. Sequence Context Corrections

A critical innovation in FINCHES is its implementation of sequence context corrections that account for local chemical environments [93]. These corrections recognize that the chemical environment of individual residues significantly influences their interaction propensities, moving beyond simple pairwise additivity assumptions that characterize many earlier approaches.

The charge weighting correction addresses the observation that clusters of like-charged residues exhibit reduced repulsion due to charge regulation effects and potential reorientation of side chains [94]. This correction is based on the physical understanding that high local charge density can lead to charge regulation through various mechanisms, including side chain reorientation, local pH shifts, and screening by mobile ions or polar groups.

For illustrative purposes, consider a sequence fragment “KKKDDD,” where the correction is calculated through the following systematic steps. The local fraction of charged residues (FCR) is calculated as the number of charged residues divided by the total number of residues in the window: FCR = 6/6 = 1.0 since all residues in this fragment are charged. The local net charge per residue (NCPR) is calculated as the absolute value of the net charge divided by the total number of residues: NCPR = |3−3|/6 = 0.0 since the net charge is zero (three positive lysines and three negative aspartates). The weighting factor is then applied as |NCPR/FCR| = |0.0/1.0| = 0.0, which effectively reduces repulsion between like charges in this balanced charged cluster, reflecting the physical reality that charge regulation can minimize electrostatic penalties in such arrangements through local charge neutralization and reorganization.

The current implementation uses static charge estimates based on physiological pH (7.4), assigning standard charges to ionizable residues (lysine and arginine +1, aspartate and glutamate −1, and histidine +0.1 to account for partial protonation). While pH-dependent protonation and deprotonation effects are not explicitly modeled through dynamic pKa calculations, the charge weighting correction partially accounts for charge regulation effects that occur when like-charged residues cluster together, providing a reasonable approximation for most physiological conditions where pH variations are typically modest.

The aliphatic weighting correction recognizes that hydrophobic residues require sufficient local density to form effective hydrophobic interfaces [95]. This correction is based on the physical principle that hydrophobic interactions become more favorable when multiple hydrophobic residues can cooperatively exclude water and form “dry” interaction surfaces. Aliphatic residues are classified into three categories based on local clustering within a window of seven residues centered on the target residue. Isolated hydrophobic residues, defined as having fewer than two hydrophobic neighbors in the window, receive no correction (1.0× multiplier) since they cannot form effective hydrophobic patches. Clustered hydrophobic residues with 2–3 hydrophobic residues in the window receive enhanced attraction (1.5× multiplier) to account for cooperative hydrophobic effects. Highly clustered hydrophobic residues with four or more hydrophobic residues in the window receive maximum enhancement (3.0× multiplier) to reflect the strong cooperative nature of extensive hydrophobic interactions.

The classification includes alanine, isoleucine, leucine, valine, phenylalanine, tryptophan, tyrosine, and methionine as hydrophobic residues based on their tendency to partition into hydrophobic environments and exclude water. The counting procedure includes the central residue in the cluster assessment, ensuring that the correction reflects the actual local hydrophobic environment experienced by each residue.

### 11.3. Mean-Field Calculation

The core calculation in FINCHES involves building a raw interaction matrix and processing it to obtain the final mean–field interaction parameter [96]. This process begins with the calculation of pairwise interaction energies between all residue types using the selected force field. The force field function returns the instantaneous potential energy (in kJ/mol) associated with specific inter-residue distances. To calculate inter-residue interaction parameters (ε), FINCHES integrates the force field function over all relevant distances, typically from the van der Waals contact distance to several times the Debye screening length for electrostatic interactions.

This integration yields a mean-field parameter that quantifies the overall attractive or repulsive nature of the interaction between residue types i and j. The integration limits are set based on the van der Waals radii (σ) of the interacting residues, ensuring that the calculation focuses on the relevant interaction range where meaningful contacts can occur. For electrostatic interactions, the upper integration limit is determined by the Debye screening length, beyond which electrostatic interactions become negligible due to the screening effect of mobile ions.

The resulting interaction matrix contains mean-field parameters for all 400 possible amino acid pairs (20 × 20), providing a comprehensive description of interaction preferences across the entire amino acid alphabet. These parameters are then used to calculate interaction energies for specific protein sequences by summing contributions from all relevant residue pairs, weighted by their sequence separation and local context corrections.

## 12. Comparative Analysis and Validation

A comprehensive comparison of computational methods reveals distinct strengths and limitations across different approaches, as summarized in Table 1. FINCHES demonstrates exceptional speed for variant analysis, requiring only 1 s per variant, while achieving a correlation coefficient of r = 0.91 for critical temperature prediction in FUS LCD systems. This combination of speed and accuracy makes it particularly valuable for high-throughput screening applications where large numbers of sequence variants must be evaluated rapidly. CALVADOS achieves slightly better accuracy (r = 0.89) for phase boundary prediction but requires 2 h per system, representing a significant computational burden for large-scale studies. All-atom molecular dynamics provides the highest structural accuracy (RMSD = 2.1 Å from experimental NMR data), but it demands 5 days per trajectory, making it impractical for large-scale studies. However, it is invaluable for a detailed mechanistic understanding of specific systems.

### 12.1. FUS Low-Complexity Domain

The FUS low-complexity domain has become a benchmark system for validating computational approaches due to its well-characterized phase separation behavior and availability of extensive experimental data [97]. This 163-residue region (amino acids 1–163) is rich in glycine, serine, glutamine, and tyrosine residues and readily undergoes liquid–liquid phase separation under physiological conditions [98]. The domain serves as an excellent test case for computational methods because its phase behavior can be precisely controlled through sequence modifications and environmental conditions.

FINCHES predicted that the wild-type FUS LCD has a strong, attractive interaction parameter (ε) value of −15.2 kJ/mol at physiological conditions [4]. This prediction captures the thermodynamic driving force for self-association that leads to phase separation. The framework correctly predicted that tyrosine-to-serine mutations would eliminate phase separation, with the ε value shifting to +8.7 kJ/mol for the 27Y→S variant [99]. This dramatic change from attractive to repulsive interactions reflects the loss of π–π interactions between tyrosine residues that are essential for FUS phase separation. Spatial interaction maps (intermaps) revealed that tyrosine-rich regions at positions 33–42 and 108–125 drive the strongest attractive interactions, providing molecular-level insights into the sequence determinants of phase separation [100].

Tesei et al. [17] performed microsecond-scale CALVADOS simulations of FUS LCD, reproducing experimental phase diagrams with quantitative accuracy. The simulations revealed that phase separation occurs through the formation of dynamic clusters stabilized by π–π interactions between tyrosine residues [101]. Critical temperatures were predicted within 2 K of experimental values, demonstrating the high accuracy achievable with properly parameterized coarse-grained models [102]. However, these simulations required substantial computational resources and expertise in molecular simulation techniques.

Palazzesi et al. [33] employed all-atom molecular dynamics (MD) to investigate FUS LCD phase separation, necessitating aggregate simulation times exceeding 100 μs across multiple systems. The simulations revealed detailed mechanisms of droplet formation, showing that tyrosine residues form transient π-stacks that nucleate liquid droplets [103]. However, the computational cost limited analysis to small systems (8–16 protein copies), preventing the direct simulation of macroscopic phase separation [104].

PSPredictor correctly classified FUS LCD as phase-separating with a confidence score of 0.94 [56]. The high score was attributed to the combination of low-complexity sequence composition and high aromatic content, which characterizes many phase-separating proteins [105]. However, the method cannot predict the effects of specific mutations without retraining on new data, limiting its utility for protein design applications [106].

### 12.2. DDX4 N-Terminal Domain

The DDX4 N-terminal domain (residues 1–113) contains a mixed charge distribution and has been extensively studied as a model system for understanding charge effects in phase separation [107,108]. This domain is particularly valuable for testing computational methods because its phase behavior can be dramatically altered by redistributing charges without changing the overall sequence composition, providing a stringent test of the methods’ ability to capture sequence-specific effects.

FINCHES analysis revealed that DDX4-NTD has a moderately attractive ε value of −8.1 kJ/mol [4]. The charge-shuffled variant, which redistributes the exact charges more evenly throughout the sequence, showed a dramatically different ε value of −18.7 kJ/mol, correctly predicting enhanced phase separation observed experimentally [109]. This prediction demonstrates FINCHES’s ability to capture the subtle effects of charge patterning on interaction strength. Spatial interaction maps identified specific regions (residues 20–35 and 65–80) as primary drivers of attractive interactions, guiding experimental mutagenesis studies [110].

Lin and Chan [111] used polymer field theory to study charge patterning effects in DDX4-NTD. The approach correctly predicted that charge segregation enhances phase separation, with calculated critical temperatures within 1 of experimental values [112]. The field-theoretic approach required 30 min of computation time compared to 1 s for FINCHES, highlighting the speed advantages of the analytical approach [113].

Brady et al. [114] performed detailed experimental characterization of DDX4-NTD phase behavior using single-molecule fluorescence experiments. These studies revealed that the wild-type protein forms dynamic droplets with internal exchange on timescales of seconds, while the charge-shuffled variant forms more stable condensates with reduced dynamics [115]. These experimental observations provide crucial validation for computational predictions and highlight the importance of dynamics in understanding IDR function.

### 12.3. p53 Transactivation Domain

The p53 transactivation domain (TAD1, residues 1–40) represents a classic example of a functional intrinsically disordered region (IDR) that undergoes disorder-to-order transitions upon binding to its targets [116,117]. This system presents a distinct type of validation challenge for computational methods, as it involves the formation of structured complexes rather than phase separation.

Rauscher et al. [32] performed extensive REMD simulations of p53 TAD1, revealing that the free protein samples are both compact and extended conformations. Upon binding to MDM2, the protein folds into an amphipathic helix [118]. The simulations accurately reproduced NMR chemical shift data and provided mechanistic insights into the folding process upon binding [119]. These detailed simulations required substantial computational resources but provided an atomic-level understanding of the binding mechanism.

FINCHES cannot directly model disorder-to-order transitions but can successfully predict attractive interactions between p53 TAD1 and MDM2 surface residues (ε = −12.4 kJ/mol) [4]. The spatial interaction map correctly identified the hydrophobic residues F19, L22, and W23 as key interaction drivers, consistent with experimental mutagenesis studies that showed these residues are essential for MDM2 binding [120]. While FINCHES cannot predict the structural details of the complex, it successfully identifies the thermodynamic driving forces for interaction.

Specialized predictors for protein–protein interactions (e.g., ANCHOR2) correctly identified the MDM2-binding region in p53 TAD1 based on sequence features [121]. However, these methods require prior knowledge of binding partners and cannot predict novel interactions, limiting their utility for discovery applications [122].

### 12.4. TDP-43 Low-Complexity Domain

The TDP-43 LCD (residues 267–414) is implicated in ALS and frontotemporal dementia and contains a conserved hydrophobic region that drives pathological aggregation [123,124]. This system provides important validation for methods aimed at understanding disease-related protein aggregation.

Lancaster et al. [70] applied PLAAC to TDP-43 LCD, identifying a prion-like region spanning residues 320–366. The method correctly predicted that ALS-associated mutations in this region increase the aggregation propensity [125]. PLAAC scores for disease mutations were significantly higher than for control variants, demonstrating the method’s ability to identify pathologically relevant sequence features [126].

FINCHES predicted that wild-type TDP-43 LCD has moderately attractive interactions (ε = −6.8 kJ/mol) but identified the conserved region as a hotspot for intermolecular contacts [4]. Phosphorylation simulations demonstrated that modification of the C-terminal serines significantly reduces attractive interactions, consistent with experimental observations that phosphorylation suppresses aggregation [127]. This demonstrates FINCHES’s utility for understanding the post-translational regulation of protein interactions.

Gruijs da Silva et al. [128] utilized CALVADOS to investigate the effects of TDP-43 phosphorylation, demonstrating that 12S→D mutations abolish phase separation. The simulations revealed that phosphomimetic mutations disrupt the delicate balance between attractive and repulsive interactions required for condensate formation [129]. These studies underscore the significance of post-translational modifications in modulating IDR interactions.

### 12.5. Speed and Scalability Analysis

Computational performance analysis reveals significant differences in scalability across methods, as detailed in Table 2. FINCHES demonstrates linear scalability with sequence length and requires only standard CPU hardware, making it accessible for routine use in any research laboratory. The method’s computational requirements scale as O(L^2^) for sequence length L when considering all pairwise interactions, but the constant factor is extremely small due to the analytical nature of the calculations.

In contrast, molecular dynamics simulations exhibit an exponential scaling with system size and require specialized computing clusters, thereby limiting their application to focused studies of specific systems. CALVADOS simulations scale quadratically with the number of proteins due to the need to calculate all pairwise interactions during the simulation. In contrast, all-atom MD simulations scale even more unfavorably, owing to the larger number of atoms and shorter timesteps required for numerical stability.

The speed advantages of FINCHES become particularly apparent for large-scale studies [130]. Analyzing all 19,702 human IDRs for phosphorylation effects required only 3.2 h using FINCHES, compared to an estimated 45 years for equivalent CALVADOS simulations or 890 years for all-atom molecular dynamics (MD) studies [131]. This dramatic difference in computational requirements makes FINCHES uniquely suited for proteome-scale analyses that would be impossible with traditional simulation approaches.

Accuracy comparisons across different prediction types reveal that different methods excel in other domains, as shown in Table 3. FINCHES achieves 87% correct classification for binary phase separation prediction and a correlation coefficient of r = 0.85 for critical temperature prediction, providing good overall performance across diverse applications.

CALVADOS achieves higher accuracy for phase separation prediction (94% vs. 87%) but at the cost of significantly longer computation times. All-atom molecular dynamics (MD) provides the highest accuracy for interface prediction (89% vs. 73%) but is limited to small systems. Machine learning methods show poor transferability to new mutations (45% accuracy), highlighting the importance of physics-based approaches for protein design applications.

## 13. Key Outputs and Interpretations

### 13.1. Mean-Field Interaction Parameter (ε)

The primary output of FINCHES is the mean-field interaction parameter ε, which quantifies the overall driving force for interaction between two sequences [4]. Negative ε values indicate net attractive interactions that favor complex formation or self-association, while positive values suggest repulsive interactions that disfavor association [132]. The magnitude of ε correlates with interaction strength, enabling quantitative comparisons between different sequence pairs and conditions [133]. This parameter provides a thermodynamic measure of interaction favorability that can be directly related to experimental observables such as binding constants and critical concentrations.

For homotypic interactions (self-association), ε values can predict the propensity for phase separation [134]. The framework has successfully reproduced experimental phase diagrams for numerous IDR systems, including FUS, TDP-43, and hnRNPA1, demonstrating that sequence-based ε calculations can capture the essential thermodynamics of protein condensation [135,136,137]. The relationship between ε values and experimental phase boundaries follows theoretical predictions from polymer physics, providing confidence in the physical basis of the projections.

### 13.2. Intermaps: Spatial Resolution of Interactions

Beyond global ε values, FINCHES generates intermaps that provide residue-level resolution of interaction landscapes [138]. These two-dimensional heat maps show which specific regions of two interacting sequences drive attractive or repulsive interactions [139]. Intermaps are calculated using a sliding window approach, where local ε values are computed for sequence segments of defined size (typically 13–31 residues) across all possible pairwise combinations [140]. This analysis reveals the spatial organization of interaction hotspots and enables the identification of specific sequence regions responsible for mediating interactions.

Representative intermaps for several well-characterized systems show clear, attractive regions corresponding to experimentally identified interaction hotspots [141]. The DDX4 N-terminal domain exhibits attractive regions that align with experimental binding studies, while charge-shuffled variants display dramatically altered interaction patterns that correlate with altered phase separation behavior [114]. These spatial predictions enable researchers to identify specific residues or regions responsible for mediating interactions, facilitating targeted mutagenesis experiments and functional studies [142]. The high spatial resolution of intermaps makes them particularly valuable for protein design applications where specific interaction interfaces need to be optimized.

### 13.3. Phase Diagram Predictions

FINCHES can predict liquid–liquid phase separation behavior by combining ε values with principles of polymer physics [143]. Using the Flory–Huggins framework adapted for protein solutions, the method generates phase diagrams showing critical temperatures and concentrations for phase separation [144]. These predictions have shown remarkable agreement with experimental measurements across diverse IDR systems, correctly capturing the effects of sequence mutations, salt concentration, and temperature changes [145]. The ability to predict complete phase diagrams from sequence information alone represents a significant advance in understanding the physical basis of protein phase separation.

## 14. Applications and Experimental Validation

### 14.1. Proteome-Scale Analysis

One of FINCHES’s most powerful applications is a large-scale analysis of interaction networks [146]. The framework has been applied to analyze all human IDRs longer than 100 amino acids, revealing global patterns in interaction propensities and identifying proteins with unusual interaction characteristics [147]. This analysis revealed that proteins with highly attractive homotypic interactions are significantly underrepresented among highly abundant cellular proteins, suggesting evolutionary pressure against promiscuous aggregation [148]. This observation offers valuable insights into the evolutionary constraints that shape protein sequence evolution and the mechanisms cells employ to prevent pathological protein aggregation.

Gene ontology enrichment analysis revealed that proteins with strong, attractive interactions are enriched in RNA processing and transcriptional regulation functions, consistent with their roles in forming membrane-less organelles, such as nuclear speckles and P-granules [149]. This large-scale analysis would be computationally prohibitive using traditional simulation approaches, but is readily achievable with FINCHES [150]. The ability to perform proteome-scale analyses opens new opportunities for understanding cellular organization and identifying novel therapeutic targets.

### 14.2. Post-Translational Modification Effects

FINCHES has proven particularly valuable for studying how post-translational modifications alter interaction landscapes [151]. Phosphorylation analysis of 19,702 human intrinsically disordered regions (IDRs) revealed distinct patterns depending on protein function [152]. RNA-binding proteins generally showed reduced homotypic interactions upon phosphorylation, consistent with phosphorylation serving as a mechanism to dissolve RNA–protein condensates [153]. In contrast, signaling proteins often showed enhanced interactions, suggesting phosphorylation-induced complex formation [154]. These findings provide molecular-level insights into how cells utilize post-translational modifications to regulate protein interactions and cellular organization dynamically.

The framework successfully predicted experimental observations for specific systems, including the dissolution of FUS condensates upon tyrosine phosphorylation and the enhanced interactions of phosphorylated transcriptional coactivators [155]. These predictions offer mechanistic insights into how cells utilize post-translational modifications to regulate protein interactions and phase behavior [156] in a dynamic manner. The rapid calculation speed of FINCHES makes it practical to analyze the effects of multiple modification sites simultaneously, enabling a comprehensive understanding of regulatory mechanisms.

### 14.3. Transcription Factor-Coactivator Interactions

FINCHES has been applied to understand the molecular basis of transcription factor activation domain function [157]. By calculating interactions between transcriptional activation domains and the structured binding domains of coactivator proteins, such as Mediator, the framework identified key sequence features that drive productive interactions [158]. The analysis revealed that effective activation domains must balance attractive interactions with coactivators against repulsive homotypic interactions that prevent self-association [159]. This balance is critical for proper transcriptional regulation, as excessive self-association can lead to the formation of inactive aggregates.

The relationship between activation domain strength and calculated interaction parameters shows that strong activation domains exhibit favorable interactions with Gal11/Med15 (negative ε values) while avoiding excessive self-association [160]. This analysis provided molecular-level insights into a decades-old puzzle in transcriptional regulation and demonstrated how FINCHES can illuminate the mechanistic basis of protein function [161]. The ability to predict transcriptional activity from sequence information has important implications for understanding gene regulation and designing synthetic transcriptional circuits.

### 14.4. Drug Discovery and Protein Design

The rapid calculation speed of FINCHES makes it suitable for screening applications in drug discovery and protein design [162]. The framework can quickly evaluate how sequence modifications affect interaction profiles, enabling the rational design of IDR variants with desired properties [163]. Several groups have used FINCHES predictions to design proteins with enhanced or reduced phase separation propensity, validating the predictive power of the approach [164]. Representative applications and validation results demonstrate the practical utility of FINCHES predictions across diverse systems, as detailed in Table 4.

The excellent agreement between FINCHES predictions and experimental observations across diverse systems demonstrates the robustness and general applicability of the approach. The method’s ability to predict mutation effects makes it particularly valuable for protein-engineering applications where specific interaction properties need to be optimized.

## 15. Limitations and Critical Assessment

### 15.1. Fundamental Assumptions and Consequences

While FINCHES represents a significant advance in IDR interaction prediction, it operates under several assumptions that limit its applicability and accuracy [166]. The most fundamental limitation is the mean-field approximation, which treats each residue as experiencing an average chemical environment rather than the specific, dynamic environments present in real proteins [167]. This approximation necessarily smooths over important heterogeneities and cannot capture cooperative effects that emerge from specific spatial arrangements of residues [168]. Real protein interactions often involve complex networks of contacts that exhibit significant cooperativity, where the formation of one contact facilitates the formation of additional contacts in the vicinity.

The framework assumes that IDR interactions can be decomposed into pairwise residue contributions, neglecting higher-order interactions that may be crucial for specificity [169]. Real protein interactions often involve complex networks of contacts that cannot be simply summed from individual residue pair contributions [170]. For example, the formation of β-sheet structures involves cooperative hydrogen bonding patterns that cannot be captured by pairwise additivity. Additionally, the current implementation cannot predict structured complexes formed by intrinsically disordered regions (IDRs), missing an important class of functional interactions where disorder-to-order transitions occur upon binding [171].

### 15.2. Temporal and Dynamic Limitations

Perhaps the most significant limitation of FINCHES is its static nature [172]. IDRs are inherently dynamic, existing as rapidly interconverting ensembles of conformations on timescales from nanoseconds to milliseconds [173]. The biological relevance of any interaction depends not only on its thermodynamic favorability but also on the kinetics of complex formation and dissociation [174]. FINCHES predictions represent equilibrium thermodynamic preferences but cannot address whether predicted interactions will occur on relevant biological timescales [175]. For example, two proteins might have favorable interaction energies but never encounter each other in the cell due to spatial separation or kinetic barriers.

The cellular environment is also highly crowded and heterogeneous, with local concentrations, pH, and ionic strength varying significantly from the bulk conditions typically assumed in calculations [176]. IDRs may experience very different chemical environments when localized to specific cellular compartments or when interacting with membranes, nucleic acids, or other cellular components [177]. FINCHES cannot account for these environmental complexities, potentially leading to predictions that fail to reflect in vivo behavior [178]. For instance, the presence of RNA can dramatically alter protein phase separation behavior through changes in effective valency and interaction strength.

### 15.3. Force Field Limitations

The accuracy of FINCHES predictions is fundamentally limited by the quality of the underlying force fields [179]. Current IDR force fields, while representing substantial improvements over earlier approaches, remain simplified representations of complex chemical interactions [180]. They may not accurately capture all aspects of aromatic–aromatic interactions, cation–π interactions, or the subtle effects of amino acid context on chemical properties [181]. For example, the strength of π–π interactions between aromatic residues can be significantly influenced by their local environment; however, current force fields use fixed parameters that may not accurately capture this variability.

The force fields used in FINCHES were primarily trained on single-chain properties, such as the radius of gyration and end-to-end distances, with intermolecular interaction parameters derived from limited experimental datasets [182]. The transferability of these parameters to the full diversity of IDR interactions found in biological systems remains an open question [183]. Additionally, the force fields do not account for sequence-specific effects that may arise from evolutionary selection for particular functional properties [184]. Proteins that have evolved specific functional interactions may exhibit sequence features that optimize their interaction properties in ways not captured by general force field parameters.

### 15.4. Experimental Validation Challenges

Validating FINCHES predictions presents significant experimental challenges [185]. Many of the systems used for validation involve artificially concentrated protein solutions, which may not accurately reflect physiological conditions [186]. Phase separation studies, although informative, often employ protein concentrations that are orders of magnitude higher than typical cellular levels [187]. The biological relevance of interactions observed under these conditions is not always clear [188]. Cellular protein concentrations are typically in the micromolar range, while many in vitro studies use millimolar concentrations to observe phase separation on experimentally convenient timescales.

Furthermore, most experimental validations focus on relatively simple, well-characterized systems [189]. The behavior of more complex, multi-domain proteins, which contain both structured and disordered regions, may not be accurately captured by current approaches [190]. The framework’s performance on proteins with multiple intrinsically disordered regions (IDRs), alternative splicing variants, or proteins undergoing complex regulatory modifications remains largely untested [191]. Many cellular proteins contain numerous functional domains that can influence each other’s behavior through allosteric mechanisms or competitive binding, effects that are difficult to capture in simplified experimental systems.

### 15.5. Comparative Limitations

Machine learning approaches face the fundamental challenge of limited training data [192]. The number of experimentally characterized IDR interactions is still relatively small compared to the diversity of possible sequences and conditions [193]. This leads to overfitting and poor generalization to new systems, particularly for sequences that differ significantly from training data [194]. The rapid growth in sequence databases far outpaces the accumulation of experimental data, creating an increasingly large gap between available sequence information and functional characterization.

Simulation-based methods, while providing detailed mechanistic information, face significant timescale limitations [195]. Most biologically relevant processes involving IDRs occur on timescales ranging from seconds to minutes, while simulations typically access timescales of microseconds to milliseconds [196]. Enhanced sampling methods help but cannot completely overcome this fundamental limitation [197]. This timescale gap means that many critical biological processes, such as stress granule assembly and disassembly, cannot be directly simulated with current computational resources.

All physics-based methods, including FINCHES, are fundamentally limited by the accuracy of underlying force fields [198]. Current IDR force fields, while much improved, still represent simplified descriptions of complex chemical interactions [199]. They may not accurately capture all aspects of aromatic–aromatic interactions, cation–π interactions, or context-dependent effects [200]. The development of more accurate force fields remains an active area of research, with ongoing efforts to incorporate quantum mechanical calculations and machine learning approaches to improve parameter accuracy.

## 16. Future Directions and Improvements

### 16.1. Integration of Multiple Approaches

Future developments should focus on integrating the strengths of different computational approaches [201]. Ensemble methods that combine FINCHES predictions with machine learning outputs and simulation results could provide more robust and accurate predictions [202]. For example, FINCHES could rapidly screen large numbers of sequences to identify promising candidates, which could then be studied in detail using more computationally intensive methods [203]. This hierarchical approach would leverage the speed of analytical methods for initial screening while providing detailed mechanistic insights through simulations of selected systems.

The integration of computational assessment tools, such as DockQv2, I-INF, and I-RMSD, into FINCHES validation protocols, could provide standardized benchmarking capabilities, making it particularly valuable for evaluating prediction accuracy against experimental complex structures. Such integration would enable a systematic comparison of different computational approaches and provide confidence measures for predictions in the absence of experimental validation. The development of standardized benchmarking protocols would facilitate method comparison and drive improvements in prediction accuracy across the field.

Enhanced environmental modeling represents a critical area for improvement [204]. This includes the explicit modeling of molecular crowding, pH variations, membrane proximity, and the presence of RNA or other cofactors that modulate IDR behavior [205]. The development of environment-specific corrections to existing methods could significantly improve biological relevance [206]. For example, incorporating the effects of RNA on protein phase separation could enable more accurate predictions of ribonucleoprotein granule formation and dynamics.

Incorporating kinetic information into prediction frameworks represents another important direction [207]. Methods that can predict not only thermodynamic favorability but also association and dissociation rates would provide more complete pictures of IDR function [208]. This might involve combining equilibrium predictions with kinetic modeling approaches or machine learning methods trained on time-resolved experimental data [209]. Understanding the kinetics of IDR interactions is crucial for predicting their biological function, as rapid dynamics are often essential for proper cellular regulation.

### 16.2. Experimental Integration

Future computational frameworks should be designed with better integration of experimental constraints in mind [210]. Methods that can incorporate data from NMR, SAXS, single-molecule fluorescence, and other biophysical techniques could provide more accurate and biologically relevant predictions [211]. The development of hybrid approaches that combine computational predictions with experimental constraints could leverage the strengths of both approaches while mitigating their limitations.

## 17. Conclusions

The landscape of computational methods for predicting IDR interactions has evolved rapidly, with each approach offering unique advantages and facing specific limitations [212]. FINCHES represents a significant advance by providing rapid, interpretable predictions based on established physical principles, making it particularly valuable for large-scale screening and hypothesis generation [4]. However, the static, mean-field nature of the approach limits its ability to capture the full complexity of dynamic IDR interactions [213].

Comparison with other methods reveals complementary strengths: all-atom simulations provide detailed mechanistic insights but are computationally prohibitive for large-scale studies [214]; machine learning approaches can capture complex patterns but suffer from limited training data and poor transferability [215]; coarse-grained simulations offer a balance between accuracy and speed but still require significant computational resources [216].

The integration of computational assessment tools for interface prediction provides additional validation capabilities that could enhance the reliability and standardization of IDR interaction predictions. The most promising direction for the field involves developing integrated approaches that combine the strengths of different methods while mitigating their limitations [217]. FINCHES excels at rapid screening and initial characterization, while more detailed methods are better suited for mechanistic studies of specific interactions [218]. Machine learning approaches may be particularly valuable for identifying complex sequence patterns not captured by current physics-based models [219].

As experimental techniques for studying IDRs continue to improve and datasets grow larger, computational methods will undoubtedly become more accurate and broadly applicable [220]. The key challenge will be developing approaches that can capture the dynamic, context-dependent nature of IDR interactions while remaining computationally tractable for the large-scale studies needed to understand these systems at the proteome level [221]. The future of the field lies in integrating multiple computational approaches with experimental data to provide a comprehensive understanding of IDR function in biological systems.

## Figures and Tables

**Table 1:** Comprehensive method comparison with specific system applications.

Method	System Studied	Prediction Type	Experimental Validation	Accuracy/Agreement	Computational Time	Reference
FINCHES	FUS LCD variants	Phase diagrams	Y→S mutations prevent LLPS	r = 0.91 for Tc prediction	1 s per variant	[4]
CALVADOS	hnRNPA1 LCD	Phase behavior	Aromatic mutant effects	r = 0.89 for phase boundaries	2 h per system	[17]
All-atom MD	p53 TAD	Binding mechanism	NMR chemical shifts	RMSD = 2.1 Å from experiment	5 days per trajectory	[32]
PSPredictor	Stress granule proteins	Binary classification	Localization experiments	85% accuracy (239 proteins)	0.1 s per protein	[56]
PLAAC	TDP-43 variants	Prion-like propensity	Aggregation assays	79% for ALS mutations	0.05 s per protein	[70]
FuzDrop	RNA-binding proteins	Droplet regions	Fluorescence microscopy	92% sensitivity	1 s per protein	[74]
AWSEM	α-synuclein	Aggregation pathway	Fiber morphology	Correct fibril structure	12 h per trajectory	[38]
Field Theory	Elastin-like polypeptides	Critical temperature	Turbidity measurements	±5 K accuracy	10 min per system	[53]

**Table 2:** Computational performance comparison.

Method	System Size	Time per Prediction	Scalability	Hardware Requirements
FINCHES	Any sequence	0.001 s	Linear with the sequence length	Standard CPU
CALVADOS	1–10 proteins	1–10 h	Quadratic with system size	GPU recommended
All-atom MD	1–5 proteins	1–100 days	Exponential with system size	Specialized clusters
PSPredictor	Single protein	0.1 s	Constant	Standard CPU
PLAAC	Single protein	0.05 s	Linear	Standard CPU
FuzDrop	Single protein	1 s	Linear	Standard CPU
Field Theory	Parameter space	10–60 min	Linear with parameters	Standard CPU

**Table 3:** Accuracy assessment across different prediction types.

Prediction Type	FINCHES	CALVADOS	PSPredictor	Field Theory	All-Atom MD
Phase Separation Binary	87% correct	94% correct	85% correct	91% correct	N/A
Critical Temperature	r = 0.85	r = 0.91	N/A	r = 0.78	N/A
Interface Prediction	73% agreement	N/A	N/A	N/A	89% agreement
Mutation Effects	82% correct	88% correct	45% (poor)	N/A	N/A

**Table 4:** Representative FINCHES applications and validation results.

System	Experimental Observation	FINCHES Prediction	Agreement	Reference
FUS LCD variants	Y→S mutations prevent phase separation	Highly repulsive ε values for Y→S	Excellent	[135]
DDX4-NTD	R2K variant shows reduced condensation	Reduced attractive interactions	Good	[130]
TDP-43 LCD	Phosphorylation suppresses aggregation	Weakened attractive interactions	Excellent	[135]
hnRNPA1 LCD	Aromatic mutations alter phase behavior	Predicted phase diagram changes	Good	[137]
Transcription factors	AD strength correlates with coactivator binding	ε values with Gal11 correlate with activity	Good	[157]
CAPRIN-1	Salt enhances phase separation	Reduced repulsion at higher salt	Good	[165]
